# Layer-selective half-metallicity in bilayer graphene nanoribbons

**DOI:** 10.1038/srep09825

**Published:** 2015-05-07

**Authors:** Gi Wan Jeon, Kyu Won Lee, Cheol Eui Lee

**Affiliations:** 1 Department of Physics, Korea University, Seoul 136-713, Republic of Korea

## Abstract

Half-metallicity recently predicted in the zigzag-edge graphene nanoribbons (ZGNRs) and the hydrogenated carbon nanotubes (CNTs) enables fully spin-polarized electric currents, providing a basis for carbon-based spintronics. In both carbon systems, the half-metallicity arises from the edge-localized electron states under an electric field, lowering the critical electric field *D*_c_ for the half-metallicity being an issue in recent works on ZGNRs. A properly chosen direction of the electric field alone has been predicted to significantly reduce *D*_c_ in the hydrogenated CNTs, which in this work turned out to be the case in narrow bilayer ZGNRs (biZGNRs). Here, our simple model based on the electrostatic potential difference between the edges predicts that for wide biZGNRs of width greater than ~2.0 nm (10 zigzag carbon chains), only one layer of the biZGNRs becomes half-metallic leaving the other layer insulating as confirmed by our density functional theory (DFT) calculations. The electric field-induced switching of the spin-polarized current path is believed to open a new route to graphene-based spintronics applications.

BiZGNRs with terminating hydrogen atoms will be referred to as *n*-biZGNRs, the number of zigzag carbon chains *n* giving the width of the ribbon^1^,^2^,^3^,^4^,^5^,^6^,^7^,^8^. BiZGNRs can be built in three different stacking forms: AA, ABα, and ABβ^7^. In AA stacking, carbon atoms in a layer locate directly above the carbon atoms in the other layer. In AB stacking (Bernal stacking in graphite), there are two types of carbon atoms, i.e., the carbon atoms in a layer are positioned above the center of the hexagons of the other layer, defining a B-sublattice, and those positioned right on top of the carbon atoms in the other layer, forming an A-sublattice^8^,^9^,^10^,^11^. The ABα and ABβ forms are discerned by their edge alignment patterns^7^,^9^,^10^,^11^. The ground state of the AA stacking has been shown to be a non-magnetic metal or an insulator with antiferromagnetic inter- and intra-layer spin alignments^6^,^7^,^12^, while that of the ABα stacking is known to be a non-magnetic insulator^9^,^10^,^12^, or an insulating state with antiferromagnetic inter- and intra-layer spin alignments^7^. In our DFT calculations without considering van der Waals forces, ABβ stacking has an insulating ground state with antiferromagnetic intralayer and ferromagnetic interlayer spin alignments (see Fig. 1), while both AA and ABα stacking forms prefer non-magnetic states^9^,^10^. The spin alignment in the ABβ stacking is similar to that in single-layer ZGNRs^13^,^14^,^15^,^16^. Studies of biZGNRs have mostly aimed at imparting metallic properties by chemical doping and controlling the interlayer distance^6^,^17^,^18^. Because of the opposite spin orientations at opposite edges in the ABβ stacking form of biZGNRs, a transversal electric field causes an electrostatic potential difference between the edge states and thus between the opposite spin channels, which led us to investigate the electric field-induced half-metallicity in the system.

A simple model for an electrostatic potential difference between edges can be introduced for the ABβ stacking form of biZGNRs^5^,^6^. Due to the interlayer interaction, the electron states localized at the edge carbon atoms in the A-sublattice are expected to have higher energies than those in the B-sublattice^6^. The energy level shifts of the band edges of the energy bands, occupied by the edge-localized electrons, under an electric field *D* tilted at an angle *θ* from the *x*-axis can be expressed in terms of the electrostatic potential energy (see Fig. 1). For the spin channels for which the band gap closes (red spins in Fig. 2).


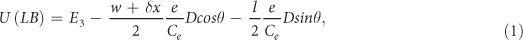



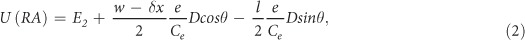



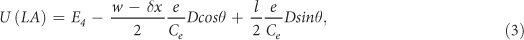



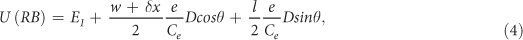


*w*, *l*, and *δx* are the nanoribbon width, the interlayer distance, and the lateral shift between the layers, respectively. The charge density localized at the edge carbon atoms is given as *e*/*C*_*e*_. *E* and *U* are the energy levels of the band edges in the absence and in the presence of electric fields, respectively, with *E*_1_ < *E*_2_ < *E*_F_ < *E*_3_ < *E*_4_ (see Fig. 2). The energy gaps *U*(LB)-*U*(RA) and *U*(LB)-*U*(RB) are smaller than the other two gaps. While *U*(LB)-*U*(RA) = *E*_3_-*E*_2_ is smaller than *U*(LB)-*U*(RB) = *E*_3_-*E*_1_ in the absence of an electric field, the electric field-induced energy level shift leading to reduction of the band gap in *U*(LB)-*U*(RB) may be greater than that in *U*(LB)-*U*(RA). From the two gaps, the electric field required for the band gap closure can be obtained as


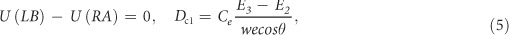



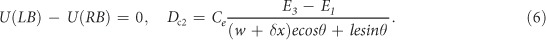


*D*_c1_ and *D*_c2_ correspond to the band gap closure in a layer and between layers, respectively, the smaller of the two being the critical electric field *D*_c_ for a band gap closure in the biZGNRs.

When *D*_c_* = D*_c2_, half-metallicity occurs between the two layers. On the other hand, when *D*_c_* = D*_c1_, only one of the layers becomes half-metallic leaving the other insulating, which would happen in wide enough biZGNRs. In this case, inversion of the electric field direction would lead to a band gap closure in the opposite spin channel, giving rise to a switching between the half-metallic and the insulating layers. Thus, our model predicts a layer-selective half-metallicity in wide enough biZGNRs and allows one to switch the path of the spin current by switching the electric field direction.

The layer-selectivity predicted by the model was confirmed by the calculations of the projected density of states (PDOS) for the edge carbon atoms in biZGNRs. Fig. 3 shows the PDOS in the upper and lower layers of 12-biZGNRs with electric fields applied along the *x*-axis. Only the PDOS of the edge carbon atoms is shown in Fig. 3, the PDOS near the Fermi level of the carbon atoms in the middle of the biZGNRs being an order of magnitude smaller than those of the edge carbon atoms. With increasing electric field, the band gap decreases in a spin channel (in red) and increases in the opposite spin channel (in blue), band gap closure occurring only in the lower layer [Fig. 3b,c]. Thus, the electric field-induced half-metallicity would occur in the lower layer only, leaving the upper layer insulating. Upon further increasing the electric field, band gap closure occurs in the upper layer as well as in the lower layer [Fig. 3d]. In other words, the layer-selective half-metallicity only occurs within a certain electric field, as confirmed by DFT calculations employing higher electric fields. When the electric field direction was reversed, the band gap was shown to close in the opposite spin channel. Thus, half-metallicity would occur only in the upper layer leaving the lower layer insulating, confirming layer-selective half-metallicity as predicted by the model above. It is to be noted that the layer-selective half-metallicity was identified only in wide *n*-biZGNRs with *n* ≥ 10.

Figure 4a to c show the band structures of a 6-biZGNR at various electric fields and tilt angles. Under an electric field applied along the *x*-axis, the spatial separation between the two spin channels leads to energy level shifts of opposite signs for them giving rise to half-metallicity (Fig. 4b). The electric field direction dependence of the electric field required for the half-metallicity, predicted by the model discussed above, was also elucidated by our DFT calculations (Fig. 4c). The *D*_c_(*θ*) were obtained from the band edges of the valence and conduction bands in *n*-biZGNRs as a function of *n* (Fig. 4d). The angle *θ*_M_ of minimum *D*_c_(*θ*) is nonzero for *n* < 10, decreasing with increasing *n* and finally becoming zero at *n* = 10, for which the layer-selective half-metallicity sets in. Fig. 4d indicates that the angle dependence of *D*_c_ is described well by our simple model introduced above. The critical electric field *D*_c_(*θ*_M_) for half-metallicity is shown to be inversely proportional to the nanoribbon width (Fig. 4e).

Our work employing a simple model based on the electrostatic potential difference between the edge carbon atoms and density functional theory calculations shows that layer-selective half-metallicity can be obtained in wide enough bilayer nanoribbons by applying electric fields. Besides, the half-metallic layer may be switched by reversing the field direction, enabling control of the spin-polarized electric current path. Thus, our results may be instrumental in opening a new field of spintronic applications utilizing carbon-based nanomaterials.

## Methods

A SIESTA version 3.2 package was employed for the DFT calculation^19^,^20^. This code adopts a localized linear combination of numerical atomic-orbital basis sets for the description of valence electrons and norm-conserving nonlocal pseudopotentials for the atomic core. The wave function is expanded with a double-ζ polarized (DZP) basis set. For the exchange-correlation potential, a generalized gradient approximation (GGA) of Perdew-Burke-Ernzerhof (PBE) form was used^21^. An energy cutoff of 210 Ry and *k*-point of 1 × 1 × 15 mesh in a Monkhorst–Pack scheme were employed for total energy convergence better than 1 meV/atom. The density of states (DOS) and the related quantities were calculated with *k* points of 1 × 1 × 1000 mesh. The atomic coordinates were optimized by using conjugated gradients method with a maximum force tolerance of 0.01 eV/Å in the absence of an electric field. The lattice parameter was set to *a* = 2.45778 Å. To simulate the external electric field, a periodic sawtooth potential was used. The supercell has 1.5-nm vacuum spacing in the *x*-direction and 1-nm vacuum spacing in the *y*-direction to avoid any intercell interactions.

In DFT codes using plane wave base sets, graphene layers have been reported not to bind with a standard GGA functional, the repulsive force being overestimated^22^,^23^. However, DFT codes using localized atomic orbitals such as SIESTA have been reported to bind graphene layers with a standard GGA functional^24^,^25^. In fact, our DFT calculations gave a well optimized interlayer distance of ~3.4 Å with or without an electric field, the layers thus holding together.

## Author Contributions

K.W.L. initiated the study and contributed to the interpretation of the data. G.W.J. performed DFT calculations and analyzed data. C.E.L. supervised the project. G.W.J., K.W.L., and C.E.L. wrote the manuscript.

## Additional Information

**How to cite this article**: Jeon, G. W. *et al*. Layer-selective half-metallicity in bilayer graphene nanoribbons. *Sci. Rep.* ****5**, 9825; doi: 10.1038/srep09825 (2015).

## Figures and Tables

**Figure 1:**
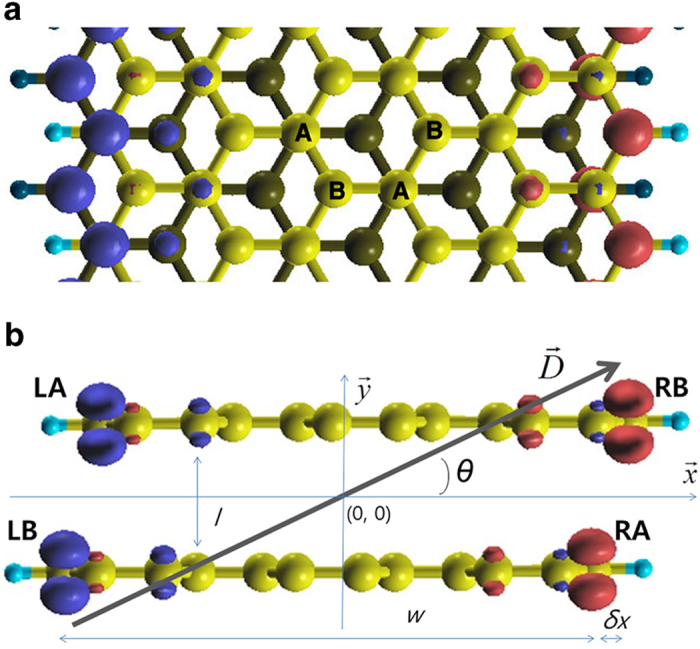
Structure and spin density of 12-biZGNR in the ABβ stacking. (**a**) Upper view of the ABβ stacking of 12-biZGNR. The yellow and cyan balls correspond to carbon and hydrogen, respectively. The red and blue spheres represent the isosurfaces of the spin densities of opposite spin orientations. (**b**) Side view of 12-biZGNR. The coordinate system used is depicted on the ABβ stacking form of 12-biZGNRs. The origin is at the center of the biZGNR with the *z*-axis parallel to the ribbon axis. The *x*-axis is parallel to the line connecting the two opposite edges and the *y*-axis is normal to the line. The symbols *w*, *l,* and *δx* represent the width of a layer, the interlayer distance, and the lateral shift between two layers. The edge carbon atoms are each labeled as LA, LB, RA, and RB, A and B representing the sublattices.

**Figure 2:**
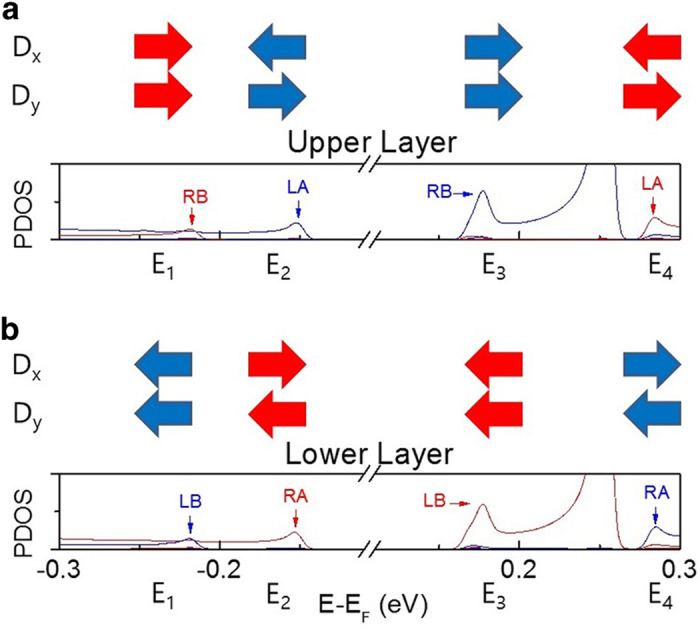
Projected density of states (PDOS) of the four edge carbon atoms. (**a**) The PDOS of the edge carbons LA and RB in the upper layer in the absence of an electric field. (**b**) The PDOS of the edge carbons LB and RA in the lower layer in the absence of electric field. The symbols *E*_1_ to *E*_4_ correspond to the energy level of the band edges, nearest to the Fermi level. The blue and red arrows indicate the energy level shift directions of the opposite spin orientations in the presence of an electric field applied along the *x*- or *y*- axes.

**Figure 3:**
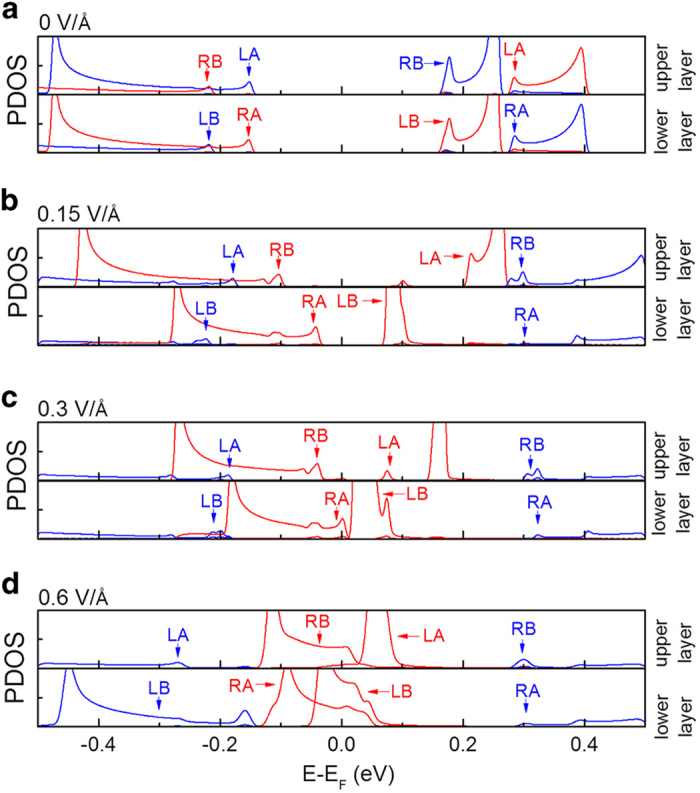
PDOS in the upper and lower layers of 12-biZGNR. (**a**) PDOS in the absence of an electric field. **(b**) PDOS in an electric field 0.15 V/angstrom along the *x*-axis. (**c**) PDOS in an electric field 0.3 V/angstrom along the *x*-axis. (**d**) PDOS in an electric field 0.6 V/angstrom along the *x*-axis. The red and blue lines correspond to the opposite spin orientations. With increasing electric field, the band gap is shown to decrease in a spin channel (in red) and increase in the opposite spin channel (in blue), band gap closure occurring only in the lower layer. Upon further increasing electric field, the band gap closes in both layers, the layer-selective half-metallicity only occurring within a certain electric field window.

**Figure 4:**
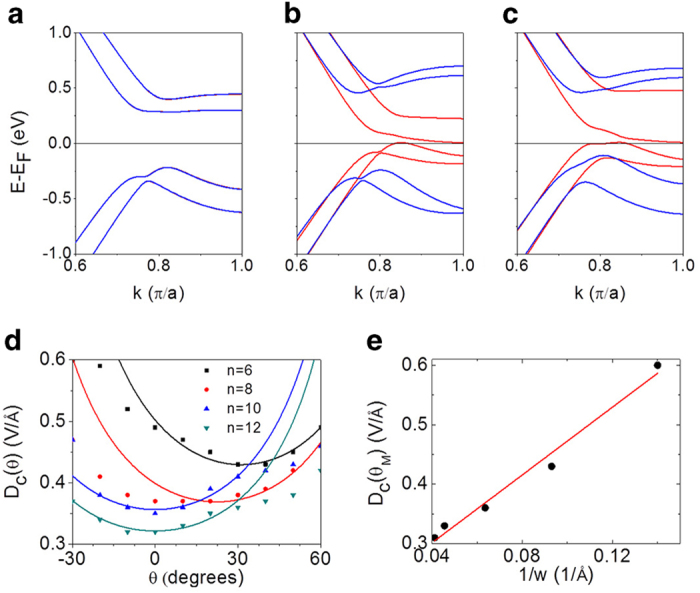
Band structures of 6-biZGNRs and the critical electric field for half-metallicity. (a) Band structure of 6-biZGNRs in the absence of an electric field. (**b**) Band structure of 6-biZGNRs in the presence of *D* = 0.49 V/angstrom applied along the *x*-axis. (**c**) Band structure of 6-biZGNRs in the presence of *D* = 0.44 V/angstrom with a tilt angle *θ* = 30 degrees. The red and blue lines correspond to the opposite spin orientations. (**d**) *D_c_(θ)* in n-biZGNRs, the solid lines representing fits to Eq. (6) for *n* = 6 and 8, and to Eq. (5) for *n* = 10 and 12, respectively. The angle *θ_M_* of minimum *D_c_(θ)* is nonzero for *n* < 10, decreasing with increasing n and finally becoming zero at *n* = 10, for which the layer-selective half-metallicity sets in. (**e**) *D_c_(θ_M_)* in n-biZGNRs as a function of the inverse of the nanoribbon width *w*. The critical electric field *D_c_(θ_M_)* for half-metallicity is shown to be inversely proportional to the nanoribbon width.
